# Rapid Adsorption of Naringin from Citrus Juice by *β*-Cyclodextrin Polymer

**DOI:** 10.3390/foods15142475

**Published:** 2026-07-13

**Authors:** Hai Tian, Shuquan Lv, Xuepei Zhou, Chaohai Pang, Bingjun Han, Yujie Feng

**Affiliations:** 1 Analysis and Test Center, Chinese Academy of Tropical Agricultural Sciences, Hainan Provincial Key Laboratory of Quality and Safety for Tropical Fruits and Vegetables, Haikou 571101, China; tianhaischolar@163.com (H.T.); pangchaohai666@126.com (C.P.); hanbingjun6868@163.com (B.H.); 2 School of Environmental and Biological Engineering, Wuhan Technology and Business University, Wuhan 430065, China; zhouxuepei6868@126.com; 3 Institute of Plant Protection, Hainan Academy of Agricultural Science, Research Center of Quality Safety and Standards of Agricultural Products in Hainan Academy of Agricultural Sciences, Haikou 571100, China

**Keywords:** *β*-CD polymer, citrus juice, bitter, naringin, adsorption mechanism

## Abstract

A *β*-cyclodextrin (*β*-CD) polymer crosslinked with tetrafluoroterephthalonitrile (1:2 molar ratio) was developed for the rapid removal of naringin, the primary bitter compound in citrus juice. The polymer achieved adsorption equilibrium within 120 s—dramatically faster than most reported naringin adsorbents—with a maximum adsorption capacity of 24.74 mg/g (Langmuir model). Kinetic data were well described by the Pseudo-second-order and Elovich models, indicating a heterogeneous, multi-site adsorption process. Isotherm analysis confirmed the coexistence of monolayer coverage and site heterogeneity (Toth model, adjR^2^ > 0.99). Under optimized conditions (adsorbent 300 mg/L, initial naringin 15 mg/L, pH 3.5), the polymer achieved an adsorption capacity of 18.84 mg/g in grapefruit juice. The adsorbed naringin was effectively eluted with 80% ethanol, and the polymer retained its original adsorption efficiency over seven consecutive cycles. Mechanistic studies (XPS, FTIR, molecular docking, and gradient elution) revealed synergistic contributions from π–π stacking, hydrogen bonding, and hydrophobic interactions. The combination of ultra-fast kinetics, good regenerability, and multi-mechanism binding makes this *β*-CD polymer a promising candidate for practical debittering of citrus juices in food processing applications.

## 1. Introduction

Citrus fruits are China’s leading horticultural crop, with the largest cultivation area, highest annual output, and strongest consumer demand in the domestic fruit industry, owing to their desirable sensory attributes including aromatic richness, pleasant flavor, and high nutritional value [1]. However, due to their short post-harvest shelf life and seasonal availability, fresh citrus fruits are commonly processed into juice. During juice production, it is essential to eliminate undesirable flavors such as bitterness, sourness, and astringency, as these off-flavors can compromise product quality, reduced consumer acceptance and diminished the economic value of the fruit-based products [2]. Among these, bitterness is predominantly attributed to compounds naringin and limonin [3,4]. Naringin is recognized as a major bitter compound in raw citrus juices; consequently, the development of effective strategies for its removal has attracted considerable research interest in the field of debittering.

Generally, physical, chemical, and biological approaches have been strategically developed to mitigate bitterness in citrus juices. The addition of syrup is a popular and convenient treatment for debittering; however, it tends to alter the original flavor profile of the juicer [5]. Optimization of juice extraction and filtration processes has shown limited effectiveness in reducing bitterness [5,6]. Biological methods, which involve the degradation of bitter compounds using microbes or enzymes, have proven effective under mild conditions. However, these methods are often costly and time-consuming, and exhibit narrow selectivity for specific bitter compounds [6,7,8,9]. Adsorption methods are frequently employed in the debittering process due to their cost-effectiveness, high efficiency, and operational simplicity. Macroporous resins, particularly non-polar types such as AB-8, PAD550, PAD600, FPX66, and LSF620, are commonly used for naringin removal [10,11]. Jiang et al. screened five macroporous resins for naringin adsorption efficiency and reported that X-5 resin achieved an adsorption capacity of 32.6 mg/g at an initial concentration at 2.7 g/L naringin [12]. XAD-7 resin removed approximately 63% of naringin, along with most off-flavor compounds, from grapefruit juice [13]. Non-ionic macroporous adsorbent resins have also been shown to reach adsorption equilibrium for naringin within 90 min [14]. Mesoporous carbon organo-clays have been explored for the effective removal of naringin [15]. Furtherly, several novel adsorbents have been synthesized to enhance naringin adsorption efficiency. Hydrophilic surface molecularly imprinted polymers achieved selective adsorption of naringin from aqueous grapefruit extract with a recovery yield of 93% [16]. Graphene oxide adsorbed 34% of naringin from lemon peel extract, along with a substantial amount of rutin and gallic acid [17]. A resin-loaded cationic hydrogel was also developed, exhibiting an adsorption capacity of 60 mg/g [18]. Despite these advances, the limited aqueous solubility of naringin continues to constrain the adsorption efficiency of most adsorbents [19].

**Scheme 1:**
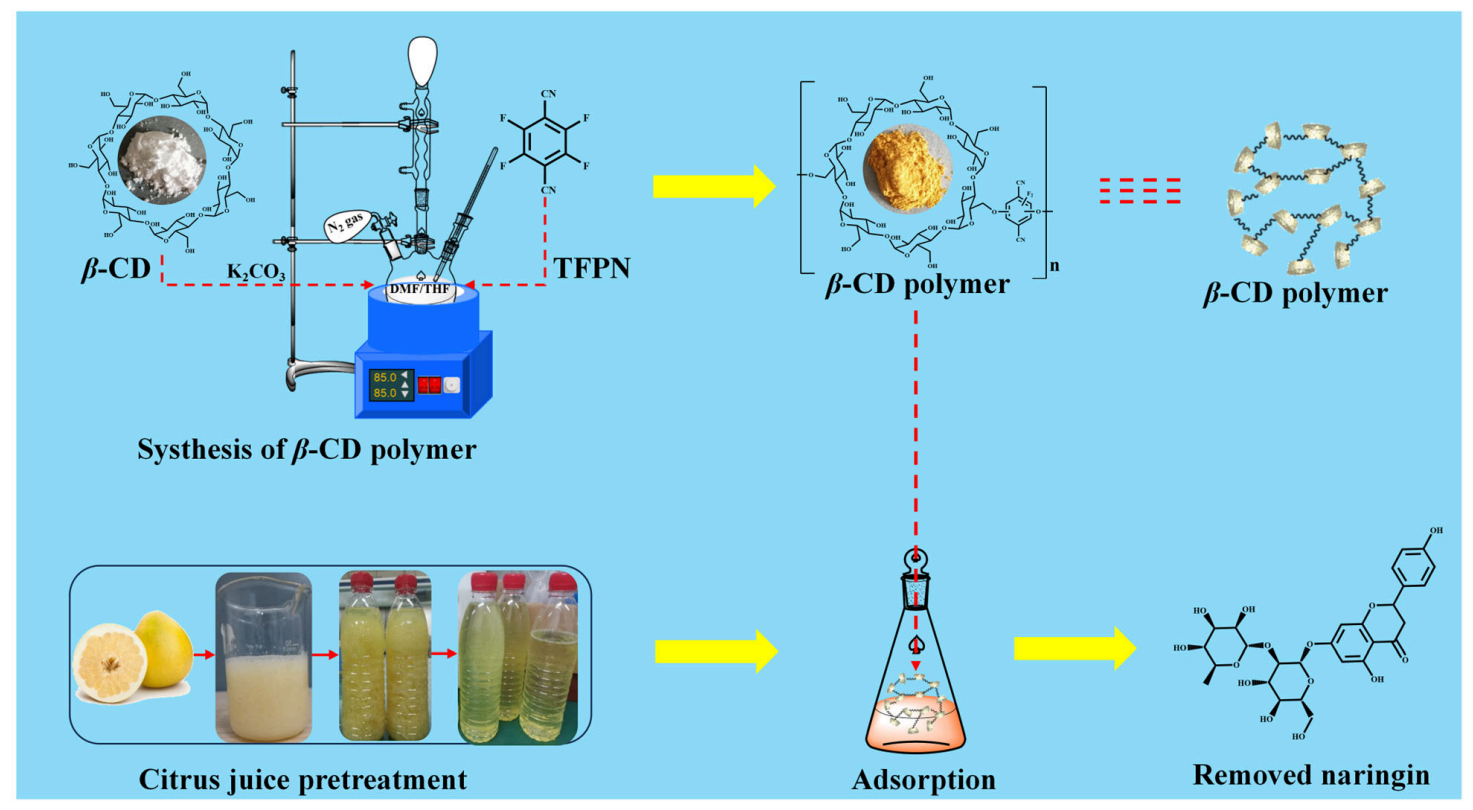
Synthesis of *β*-CD polymer and its removing naringin.

Recently, functional polymeric materials have attracted increasing attention in food processing and preservation applications [20,21]. Cyclodextrins (CDs) are cyclic α-1,4-D-glucopyranose oligosaccharides that form truncated cone-shaped macrocycles. Their amphiphilic structure, featuring a hydrophilic exterior decorated with hydroxyl groups and a hydrophobic internal cavity, facilitates host–guest complexation through hydrophilic–hydrophobic interactions [22]. Leveraging these structural features, CDs are frequently employed to enhance the solubilization of poorly water-soluble compounds and to mask undesirable flavors in aqueous solutions [23,24]. For instance, *β*-CD complexation has been reported to increase the aqueous solubility of naringin by 15-fold compared to its free form. [25]. Although the inclusion complexation of naringin with *β*-CD monomers has been extensively studied for solubility enhancement and bitterness masking [26], the reversibility of such complexes raises a concern: naringin may be released from the *β*-CD cavity over time, potentially undermining the debittering effect during extended juice storage. In contrast, crosslinked CD polymers are widely applied in environmental fields [27,28]. However, their application for the adsorptive removal of naringin from real citrus juices remains largely underexplored. In this study, three molar ratios of CD crosslinking with tetrafluoroterephthalonitrile (TFPN) were synthesized for *β*-CD polymer; among them, the polymer prepared with a 1:2 molar ratio of *β*-CD to TFPN polymer exhibited remarkable adsorption efficiency toward naringin in aqueous solution. Furtherly, this polymer could effectively remove naringin from grapefruit juice, demonstrated good reusability, and allowed elucidation of the underlying adsorption mechanism (Scheme 1).

## 2. Methods and Materials

### 2.1. Materials and Reagents

*β*-CD, TFPN, naringin (≥99%), tetrahydrofuran (THF), N, N-Dimethylformamide (DMF), K_2_CO_3_ and ethanol were obtained from Macklin Biochemical Technology Co., Ltd. (Shanghai, China). All reagents were of analytical grade.

### 2.2. Synthesis and Screening of β-CD Polymers for Adsorption Efficiency

The *β*-CD polymer was synthesized via Williamson ether synthesis. A three-neck flask equipped with a spherical condenser, magnetic stirrer, and electric heating mantle was used for the polymerization. Specifically, *β*-CD was dissolved into DMF-THF solvent (4:1, *v*/*v*), and one molar equivalent of TFPN was added to 2-, 4- and 6-fold molar equivalents of *β*-CD, respectively, with K_2_CO_3_ as catalyst. The reaction was carried out at 85 °C for 24 h under stirring at 200 rpm to obtain 1:2, 1:4, and 1:6 *β*-CD polymer. After the reaction, the mixture was allowed to cool to room temperature, and the resulting polymer was collected by filtration, sequentially washed with HCl-acidified aqueous solution, deionized water, and ethanol, and finally dried in an oven at 50 °C for 6 h.

Tannic acid, citric acid and naringin were selected to represent astringent, acidic and bitter flavors, respectively, for evaluating the adsorption efficiency of *β*-CD polymers. Batch adsorption experiments for naringin were conducted by adding 500 mg/L of the *β*-CD polymer to 25 mg/L naringin solution under agitation at 200 rpm for 2 h. After filtration through PVDF membranes (0.45 μm, Durapore^®^, Merck KGaA, Darmstadt, Germany), the residual naringin concentrations were determined using a UV–vis spectrophotometer at 280 nm based on calibration curves. The adsorption rate, adsorption kinetics (q_t_) and adsorption equilibrium (q_e_) were calculated using Equations (1)–(3).

(1) $$Adsorption\;rate=\frac{C_{0}-C_{t}}{C_{0}}100\%$$

(2) $$q_{t}=\frac{C_{0}-C_{t}}{m}v$$

(3) $$q_{e}=\frac{C_{0}-C_{e}}{m}v$$

where C_0_, C_t_, and C_e_ (mg/L) respectively represent the initial, given and equilibrium concentrations, v (L) is the volume of the solution and m (g) is the mass of the adsorbent. q_t_ or q_e_ (mg/g) represents the mass of adsorbed analyte per unit adsorbent mass at specific time or equilibrium. Data represent triplicate measurements, and their standard deviation was marked by error bars.

### 2.3. Characterization of β-CD Polymer

A comprehensive physicochemical characterization of *β*-CD polymer was conducted through a multidisciplinary analytical approach. High-resolution microstructural evaluation revealed morphological details using scanning electron microscopy (SEM, Zeiss Sigma 300, Carl Zeiss Microscopy GmbH, Oberkochen, Germany) coupled with transmission electron microscopy (TEM, JEM1400plus, JEOL Ltd., Tokyo, Japan) for nanoscale ultrastructure visualization. The chemical architecture was deciphered through complementary analytical approaches combining Fourier-transform infrared spectroscopy (FT-IR, Shimadzu Corp., Kyoto, Japan) with X-ray photoelectron spectroscopy (XPS, Thermo Scientific K-Alpha, Waltham, MA, USA) for elemental composition verification. Structural parameters including Brunauer–Emmett–Teller (BET) surface area, Barrett–Joyner–Halenda (BJH) pore size distribution, and total pore volume were systematically quantified using nitrogen physisorption porosimetry (Micromeritics ASAP 2020/2460, Norcross, GA, USA). Thermal stability profiles were evaluated through thermogravimetric analysis (PerkinElmer STA 6000, Waltham, MA, USA) under inert atmosphere, employing a controlled thermal regime from ambient conditions to 800 °C (10 °C/min heating rate) with continuous mass loss monitoring.

### 2.4. Adsorption Models

The adsorption kinetics were investigated using Pseudo-first-order (PFO), Pseudo-second-order (PSO) and Elovich models, as described in Equations (4)–(6), to evaluate adsorption kinetics onto *β*-CD polymer [29].

(4) $$q_{t}=q_{e}(1-e^{-k_{1}t})$$

(5) $$q_{t}=\frac{q_{e}^{2}k_{2}t}{1+q_{e}k_{2}t}$$

(6) $$q_{t}=\;\frac{1}{\beta }ln(\alpha \beta t+1)\;$$

In these formulations, *q_t_* (mg/g) and *q_e_* (mg/g) maintain dimensional consistency with preceding Equations (2) and (3). The PFO model features *k*_1_ (s^−1^), while the PSO employs *k*_2_ (g/(mg·s)). The Elovich equation introduces two distinctive parameters: α (mg/(g·s)) denotes initial rate coefficient, while *β* (g/mg) represents surface coverage and activation energy constant.

Three adsorption isotherm models were employed to interpret the equilibrium data: Langmuir (Equation (7)), Freundlich (Equation (8)), and Toth (Equation (9)) [30,31].

(7) $$\;q_{e}=\frac{q_{m}K_{L}C_{e}}{1+K_{L}C_{e}}$$

(8) $$q_{e}=K_{F}C_{e}^{\frac{1}{n}}$$

(9) $$q_{e}=\frac{K_{T}C_{E}}{{{(\alpha }_{T}+C_{e}^{Z})}^{1/Z}}$$

In these formulations, *C_e_* (mg/L) and *q_e_* (mg/g) represent the same values as those mentioned in the Equation (3). The Langmuir model parameters include *q_m_* (maximum adsorption capacity in mg/g) and *K_L_* (Langmuir constant in L/mg). The Freundlich equation incorporates *K_F_*, an adsorption energy constant (mg·g^−1^(L·mg)^−1/n^), along with adsorption intensity *n*. The Toth isotherm introduces three characteristic parameters: *K_T_* (constant in mg/g), *a_T_* (Toth constant in mg^z^·L^−z^), and *z* (heterogeneity index quantifying surface irregularity).

The model adequacy evaluation employed two statistical metrics: the correlation of determination (*R*^2^) and adjusted coefficient of determination (*adjR*^2^), mathematically formalized in Equations (10) and (11).

(10) $$R^{2}=(\frac{\sum_{i}^{n}{\left(q_{i,exp-}{\bar{q}}_{i,exp}\right)}^{2}-\sum_{i}^{n}{\left(q_{i,exp-}{\bar{q}}_{i,model}\right)}^{2}}{\sum_{i}^{n}{\left(q_{i,exp-}{\bar{q}}_{i,exp}\right)}^{2}})$$

(11) $$adjR^{2}=1-(1-R^{2})\cdot (\frac{n-1}{n-p-1})$$

The adsorption quantification framework employed three critical parameters: experimental measurements (*q_exp_*, mg/g), model predictions (*q_cal_*, mg/g), and their arithmetic mean (*q_mean_*, mg/g), where n represents the number of the dataset and *p* corresponds to model parameters.

### 2.5. Naringin Adsorption in Grapefruit Juice

To optimize the adsorptive performance of *β*-CD polymer, critical operational variables including adsorbent dosage, initial naringin concentration, and solution acidity were systematically investigated using grapefruit juice as the model matrix. Grapefruit pulp and exocarp were coarsely chopped, mixed with deionized water (1:8, *w*/*v*), and homogenized. The resulting mixture was then frozen at −20 °C for one week. After thawing, a flocculent precipitate formed from the nanoemulsion-like raw juice, and the clarified juice was readily obtained by vacuum filtration for subsequent adsorption experiments. First, five dosages of *β*-CD polymer ranging from 0.1 to 0.5 mg/L were tested for their adsorption efficiency in grapefruit juice containing 12 mg/L naringin. Next, the grapefruit juice was diluted to five initial concentrations, containing 5, 7.5, 10, 12.5 and 15 mg/L naringin, and the adsorption efficiency was examined using a polymer dosage of 300 mg/L. Finally, the effect of pH on adsorption was evaluated by adjusting the grapefruit juice (containing 12 mg/L naringin) to five different pH levels ranging from 3.0 to 5.0, which covers the typical natural pH range of grapefruit juice. All experiments were conducted in triplicate at 25 °C and 200 rpm for 10 min. Based on the above results, response surface optimization (RSM) was adopted to further optimize the adsorption conditions (Details in Supplementary Information, Section S1, Table S1).

### 2.6. Taste Characteristics Determination by Electronic Tongue Assessment

Under the optimized adsorption conditions established above, both the control and treated grapefruit juice samples were analyzed using a Taste-Sensing System SA402B Plus (Intelligent Sensor Technology Co., Ltd., Atsugi, Japan). Four types of detection sensors were employed: the C00 sensor for bitterness and aftertaste-B, the AN0 sensor for B-bitterness2, the BT0 sensor for H-bitterness, and the AE1 sensor for astringency and aftertaste-A. Each sample was measured in triplicate, and the mean value was calculated for further analysis.

### 2.7. Regeneration and Recycling

To identify a suitable regenerant, 10 mg of naringin-saturated *β*-CD polymer was separately desorbed with 2 mL of various eluents, including 0.1 M HCl, 0.1 M NaOH, 80% ethanol, and 50% methanol, at 25 °C and 200 rpm. The recycling performance of the regenerated *β*-CD polymer was then evaluated under the following conditions: initial naringin concentration of 15 mg/L, temperature 25 °C, agitation speed 140 rpm, contact time 2 min, and pH 3.5.

## 3. Results and Discussion

### 3.1. Screening Adsorptive Efficiency of Three β-CD Polymers

**Figure 1:**
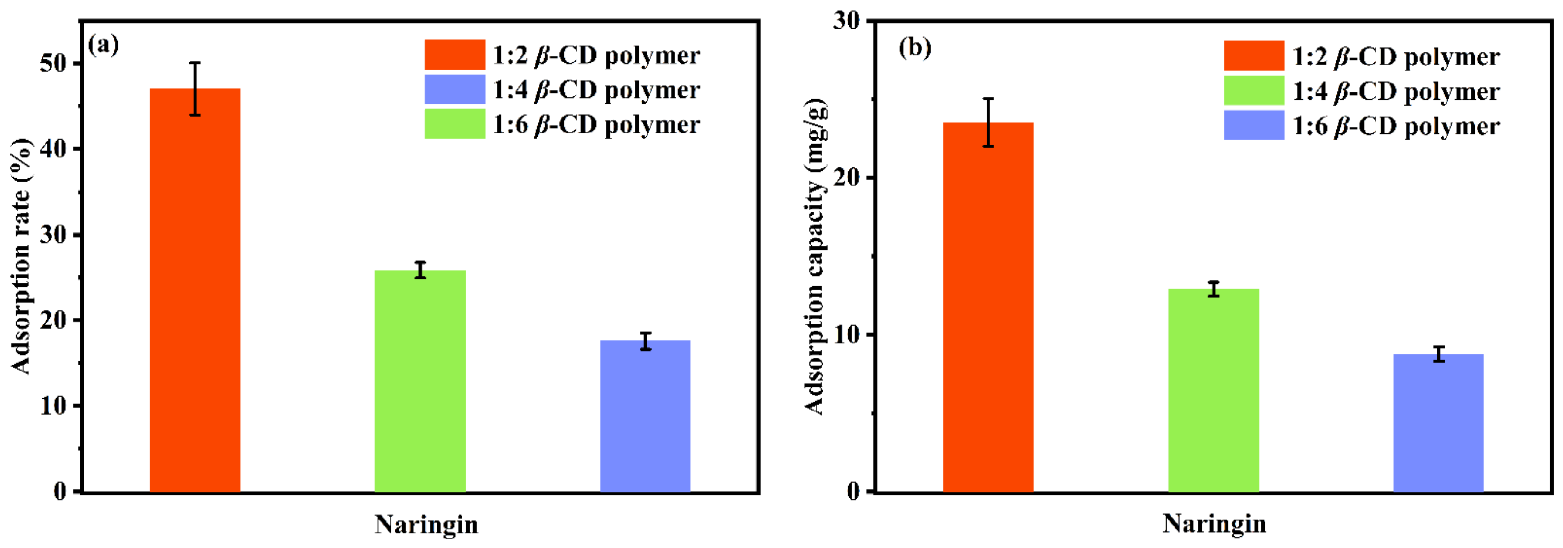
Adsorption efficiency of three *β*-CD polymers toward naringin (adsorbent dosage 500 mg/L, concentration 25 mg/L, 200 rpm, 2 h), (**a**) adsorption rate, (**b**) adsorption capacity.

Three *β*-CD polymers with different crosslinking ratios were synthesized and evaluated for their adsorption efficiency toward naringin, with the results presented in Figure 1. Among them, the 1:2 *β*-CD polymer exhibited the superior adsorption performance, achieving a removal rate of 47.04% and an adsorption capacity of 23.52 mg/g. Additionally, this polymer also showed best adsorption efficiency toward tannic acid and citric acid (Figure S1). Altogether, the 1:2 *β*-CD polymer was selected as the optimal adsorbent for subsequent naringin adsorption.

### 3.2. Physicochemical Properties of β-CD Polymer

**Figure 2:**
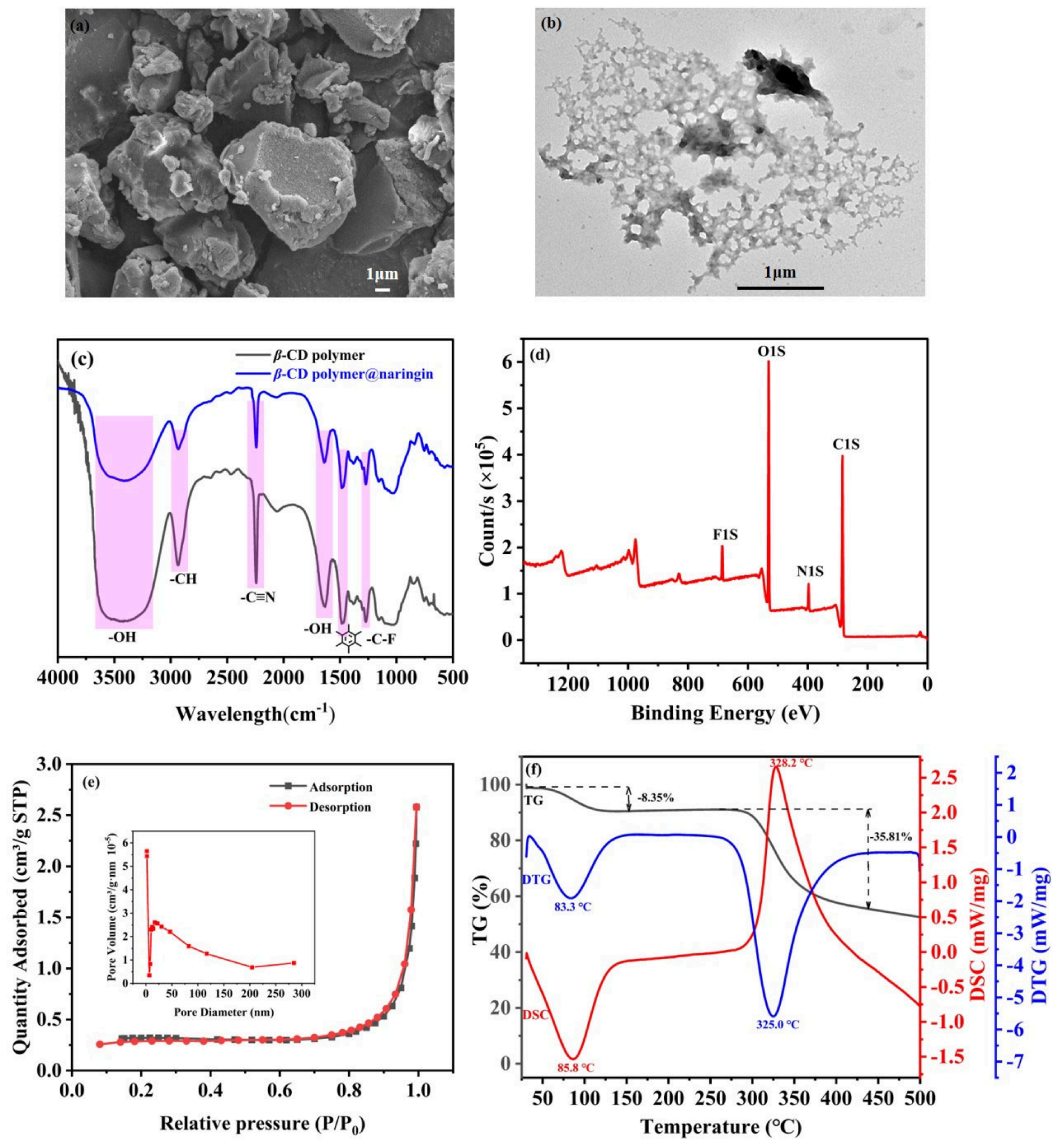
Characterization of *β*-CD polymer. (**a**) SEM image at 1 μm, (**b**) TEM image at 1 μm, (**c**) FT−IR spectra; (**d**) total survey scans of XPS spectra, (**e**) N_2_ adsorption−desorption isotherms (inner pore size distribution curve), (**f**) thermogravimetric analysis showing TG/DTG curves.

The 1:2 *β*-CD polymer, orange insoluble powder with a yield of around 25% was obtained and selected for further characterization. The morphology and surface structures were examined by SEM (Figure 2a), which revealed irregularly shaped small particles resembling pieces of broken sponge. The TEM image further showed that the *β*-CD polymer possesses numerous porous microstructures (Figure 2b). FT-IR analysis (Figure 2c) confirmed the structural integrity of *β*-CD polymer. Characteristic absorption bands of *β*-CD were well preserved in the polymeric matrix, including broad -OH stretching vibrations at 3400 cm^−1^, a hydroxyl deformation band at 1640 cm^−1^, and aliphatic -CH_2_ antisymmetric stretching at 2926 cm^−1^ [32]. Notably, three new absorption peaks appeared at 2250 cm^−1^ (C≡N triple bond stretching), 1480 cm^−1^ (aromatic C=C skeletal vibrations in para-substituted benzene rings), and 1270 cm^−1^ (C-F bond stretching), which are consistent with the TFPN moieties in the polymer network [33]. XPS spectra (Figure 2d) revealed characteristic signals of C1s, O1s, N1s and F1s, with relative contents of 53.92%, 29.62%, 12.22% and 4.25%, respectively. The BET surface area of the *β*-CD polymer was determined to be 1.0594 m^2^/g, with a micropore area of 0.9665 m^2^/g. Pore structure analysis indicated a total volume of 0.0034 cm^3^/g and a micropore volume of 0.0005 cm^3^/g, along with an average pore diameter of 12.9742 nm, indicating a predominantly mesoporous structure (Figure 2e). TGA analysis (Figure 2f) demonstrated thermal stability up to approximately 300 °C, with the initial mass loss between 50 °C and 125 °C due to moisture within the polymer matrix.

### 3.3. Adsorption Isotherms and Adsorption Kinetics

**Figure 3:**
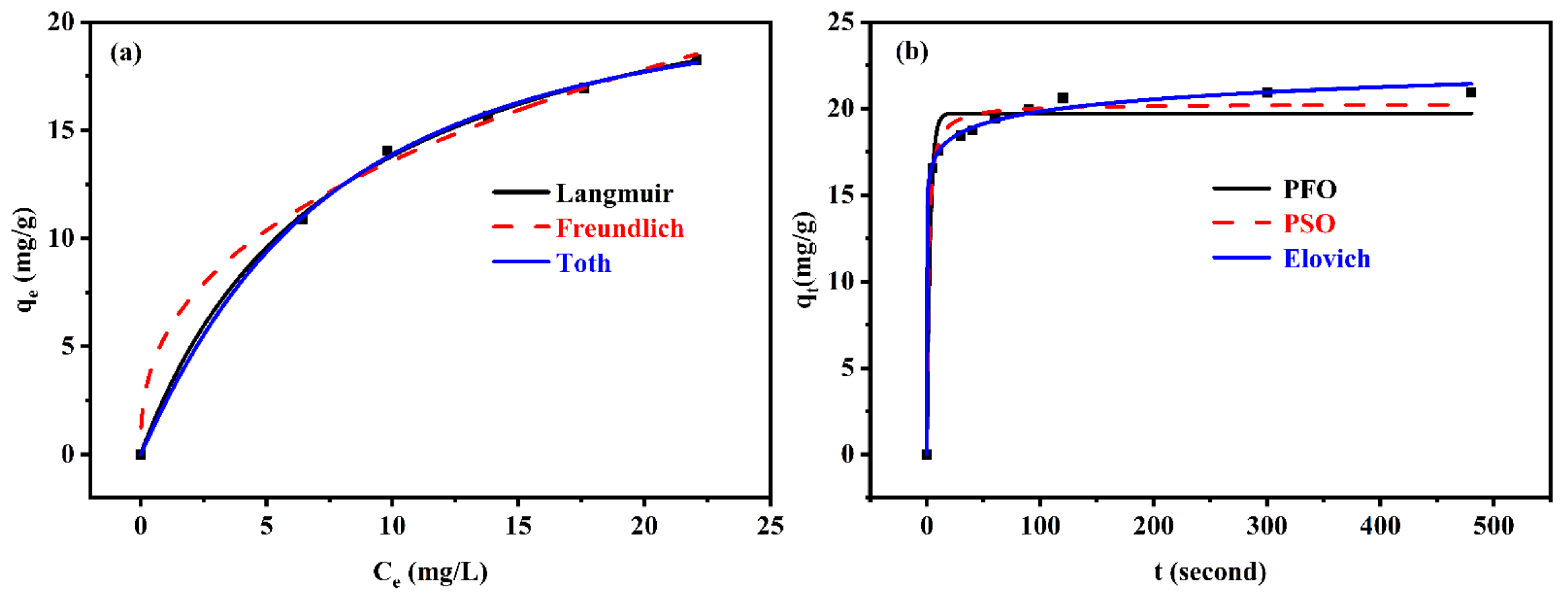
Adsorption isotherm and dynamics of *β*-CD polymer toward naringin. (**a**) Adsorption isotherm, (**b**) adsorption kinetics.

The equilibrium adsorption behavior was modeled using Langmuir, Freundlich, and Toth isotherm equations, with experimental data and fitting curves presented in Figure 3a and corresponding parameters summarized in Table 1. Adsorption experiments were conducted at five initial naringin concentrations (10, 15, 20, 25, and 30 mg/L) under standardized conditions: adsorbent dosage 200 mg/L, pH 7.0, agitation speed of 200 rpm, temperature of 25 °C, and contact time of 10 min. Among the three isotherm models, the Langmuir and Toth isotherms models provided superior fits at lower adsorbate concentrations, as judged by adjusted correlation coefficient (adjR^2^). The Langmuir model suggested that adsorption sites on the *β*-CD polymer were energetically homogeneous and that the adsorption process followed a monolayer coverage mechanism [34]. The Toth model effectively reflected that the *β*-CD polymer surface was heterogeneous in nature, and competitive sites existed during the adsorption process [35]. The excellent model fit (R^2^ > 0.99) thus confirms the energetically heterogeneous nature of the PFTN-crosslinked *β*-CD polymer surface, consistent with reports on hypercrosslinked polymeric resins [36]. This heterogeneity arises from the coexistence of multiple site types—hydrophobic CD cavities, aromatic PFTN moieties, and polar hydroxyl groups—creating a broad energy distribution. The presence of competitive sites is further supported by the Toth model’s ability to describe systems where multiple interactions coexist during adsorption, validating its appropriateness for this complex crosslinked polymer system [37].

Adsorption kinetic experiments were conducted under the following conditions: adsorbent dosage of 200 mg/L, initial naringin concentration of 10 mg/L, pH 7.0, agitation speed of 200 rpm and temperature of 25 °C. The adsorption capacity was measured at various time intervals, including 0, 5, 10, 30, 40, 60, 90, 120, 300, and 480 s. Stunningly, the adsorption equilibrium time was achieved within 120 s, and 92.87% of adsorption equilibrium capacity was already attained within the first 60 s. Furthermore, three kinetic models including PFO, PSO and Elovich were fitted to the experimental data by time (s) versus adsorption capacity (mg/g). The fitting results are presented in Figure 3b, and the corresponding kinetic parameters are summarized in Table 2. Elovich and PSO models exhibited preferable fitting based on the adjusted correlation coefficient (adjR^2^), further indicating that naringin adsorption onto the *β*-CD polymer predominantly involved a heterogeneous system with multiple active sites [38,39,40]. The Elovich model corroborates this interpretation from a kinetic perspective. Originally developed for describing adsorption on energetically heterogeneous surfaces, the Elovich equation assumes that the activation energy varies with surface coverage—a behavior characteristic of multi-site systems. In the present study, the superior fit of the Elovich model (R^2^ > 0.99) confirms that naringin adsorption proceeds via multiple active sites on the polymer surface, consistent with the chemically diverse environment created by the coexisting hydrophobic cavities, aromatic rings, and hydroxyl groups. Such a complex surface is precisely the type of system for which the Elovich equation was originally designed, further validating its applicability to this crosslinked polymer network.

Notably, many rigidly crosslinked *β*-CD polymers have achieved adsorption uptake within short periods [48,49]. The rigid PFTN crosslinker expands the polymer network into a highly porous structure with wide mass-transfer channels, which substantially promotes intraparticle diffusion; pore diffusion is the rate-limiting step in cyclodextrin-based adsorbents [50]. Rigid crosslinking strategies have been shown to significantly enhance diffusivity—for instance, phytic acid-based crosslinking tripled the diffusion rate of the flavonoid rutin compared with conventional non-porous *β*-CD beads [51]. In our system, this reduced diffusion resistance enables naringin to access the hydrophobic cavities rapidly, allowing the inclusion process to reach equilibrium within 120 s. In contrast, many flexible-crosslinker *β*-CD polymers suffer from low porosity and poor adsorption performance [52]; the rigid PFTN crosslinker thus offers a clear structural advantage, analogous to the rigid strategy reported by Qiao et al. [51], by enhancing both accessibility and kinetics.

### 3.4. Applicability in Naringin Removal from Grapefruit Juice

#### Influencing Factors of Adsorption Efficiency

**Figure 4:**
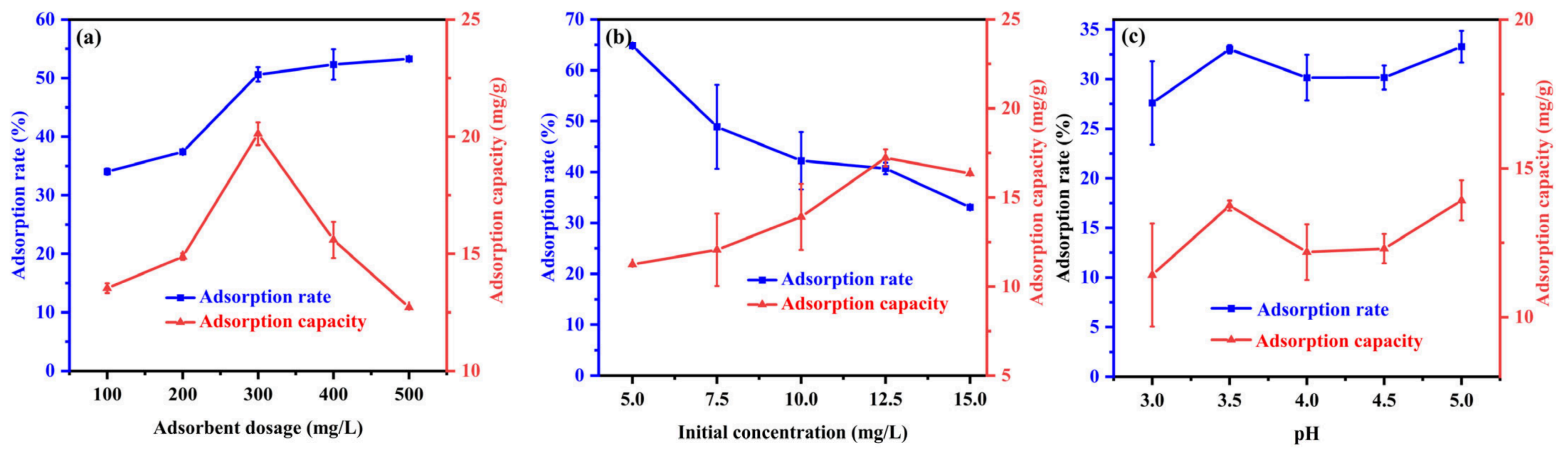
Optimization of *β*-CD polymer adsorption for naringin using three factors. (**a**) Adsorbent dosage, (**b**) initial concentration, and (**c**) pH.

The adsorption efficiency of the *β*-CD polymer toward naringin in grapefruit juice was evaluated in terms of adsorption rate and capacity, with respect to three key factors: adsorbent dosage, initial naringin concentration, and pH. As shown in Figure 4a, when the polymer dosage increased to 300 mg/L, the adsorption rate reached 50.64% with a corresponding capacity of 20.47 mg/g. However, further increasing the dosage to 500 mg/L resulted in a slightly higher adsorption rate (53.30%) but a markedly lower capacity (12.71 mg/g). The effect of initial naringin concentration was also examined (Figure 4b). The adsorption capacity reached a maximum of 17.22 mg/g at an initial concentration of 12.5 mg/L, whereas the adsorption rate consistently declined with increasing initial concentration. Generally, higher adsorbent dosage or higher initial concentration would be expected to improve adsorption efficiency. However, the observed deviations from this trend are largely attributable to competing substances present in grapefruit juice that exhibit stronger affinity for the *β*-CD polymer, thereby diminishing the effective adsorption of naringin. In addition, the adsorption efficiency exhibited two distinct peaks at pH 3.5 and 5.0 (Figure 4c). This pH-dependent behavior can be explained by the influence of acid–base conditions on the dissociation state of naringin, which in turn affected its interactions with the *β*-CD polymer [53].

**Figure 5:**
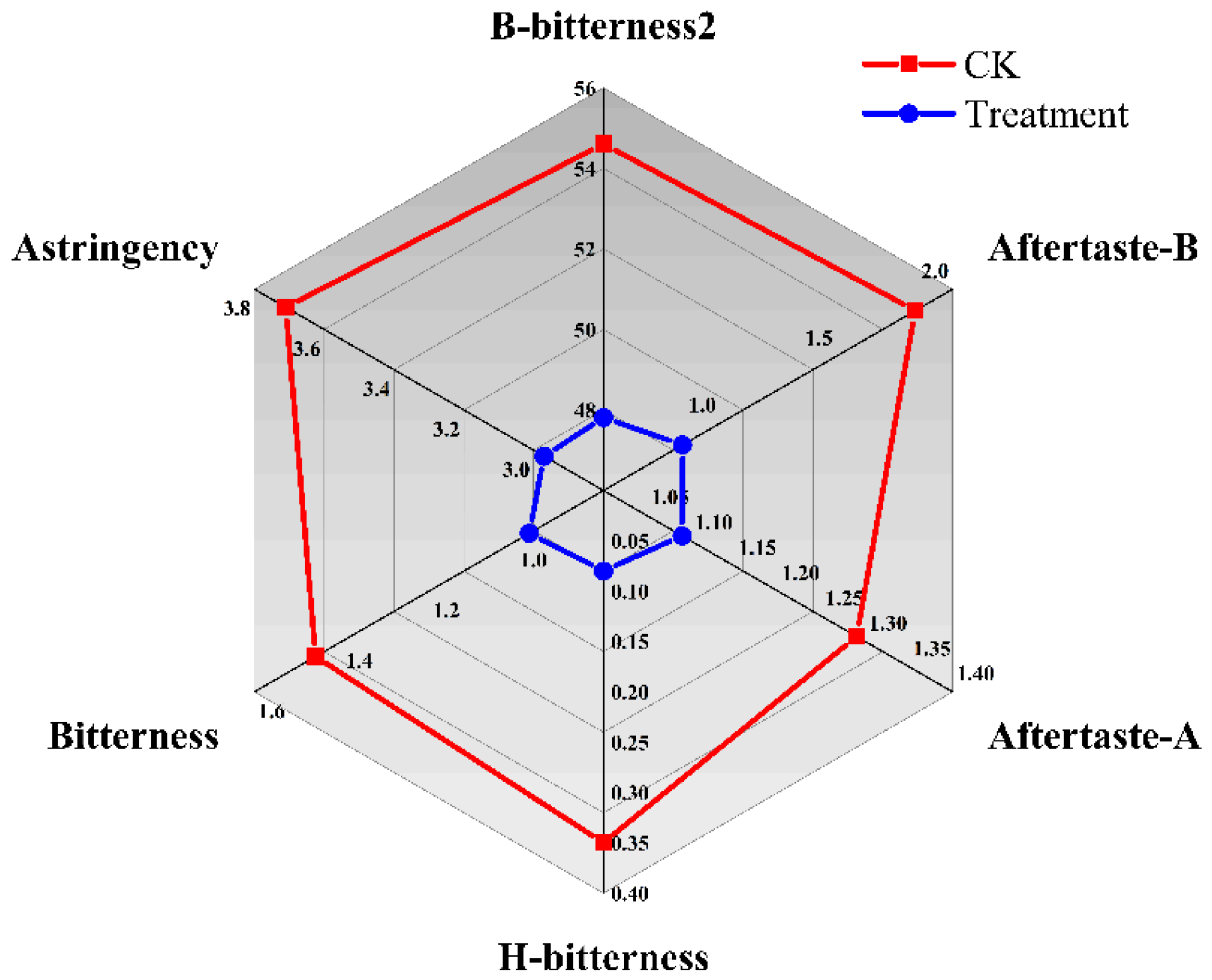
Electronic tongue radar map of adsorptive bitterness.

Design response surface methodology (RSM) based on a Box–Behnken design was employed using Design-Expert software v13 to further optimize the adsorption conditions (details in Supplementary Information, Section S2, Figures S2 and S3, Tables S2 and S3). The polymer achieved a maximum adsorption capacity of 18.84 mg/g for naringin in grapefruit juice under the following optimized conditions: adsorbent dosage of 300 mg/L, initial naringin concentration of 15 mg/L, and pH 3.5. The taste profile of the juice before and after adsorption was evaluated using an electronic tongue under these optimized conditions. As shown in Figure 5, the intensities of bitterness (acid bitterness), H-bitterness (base salt bitterness), B-bitterness2 (alkaline bitter aftertaste), and aftertaste-B (acid bitter aftertaste), as well as astringency and aftertaste-A (astringent aftertaste), were all significantly reduced after treatment with the *β*-CD polymer.

### 3.5. Recycling Performance of β-CD Polymer

**Figure 6:**
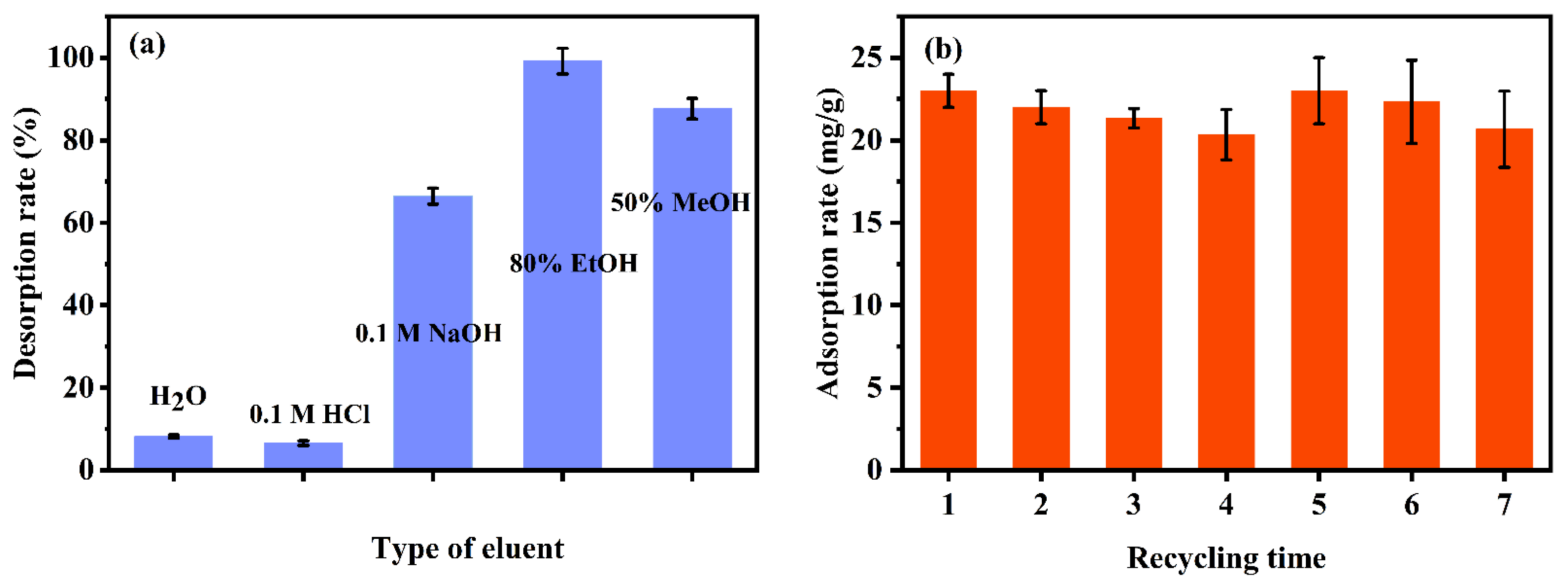
Screening eluent for *β*-CD polymer of saturated-adsorption naringin (**a**) and reusability of *β*-CD polymer toward naringin (**b**).

Five different eluents were evaluated for the regeneration of the naringin-saturated *β*-CD polymer, as shown in Figure 6a. A desorption rate exceeding 70% was achieved using 0.1 M NaOH, 50% methanol, and 80% ethanol. Notably, nearly 100% desorption was attained with 80% ethanol, which was therefore selected as the optimal eluent for subsequent recycling studies. As presented in Figure 6b, the adsorption capacity remained stable within the range of 20–23 mg/g over consecutive cycles, indicating that the *β*-CD polymer possesses good reusability and maintains its adsorption performance after regeneration.

### 3.6. Potential Adsorption Mechanism

**Figure 7:**
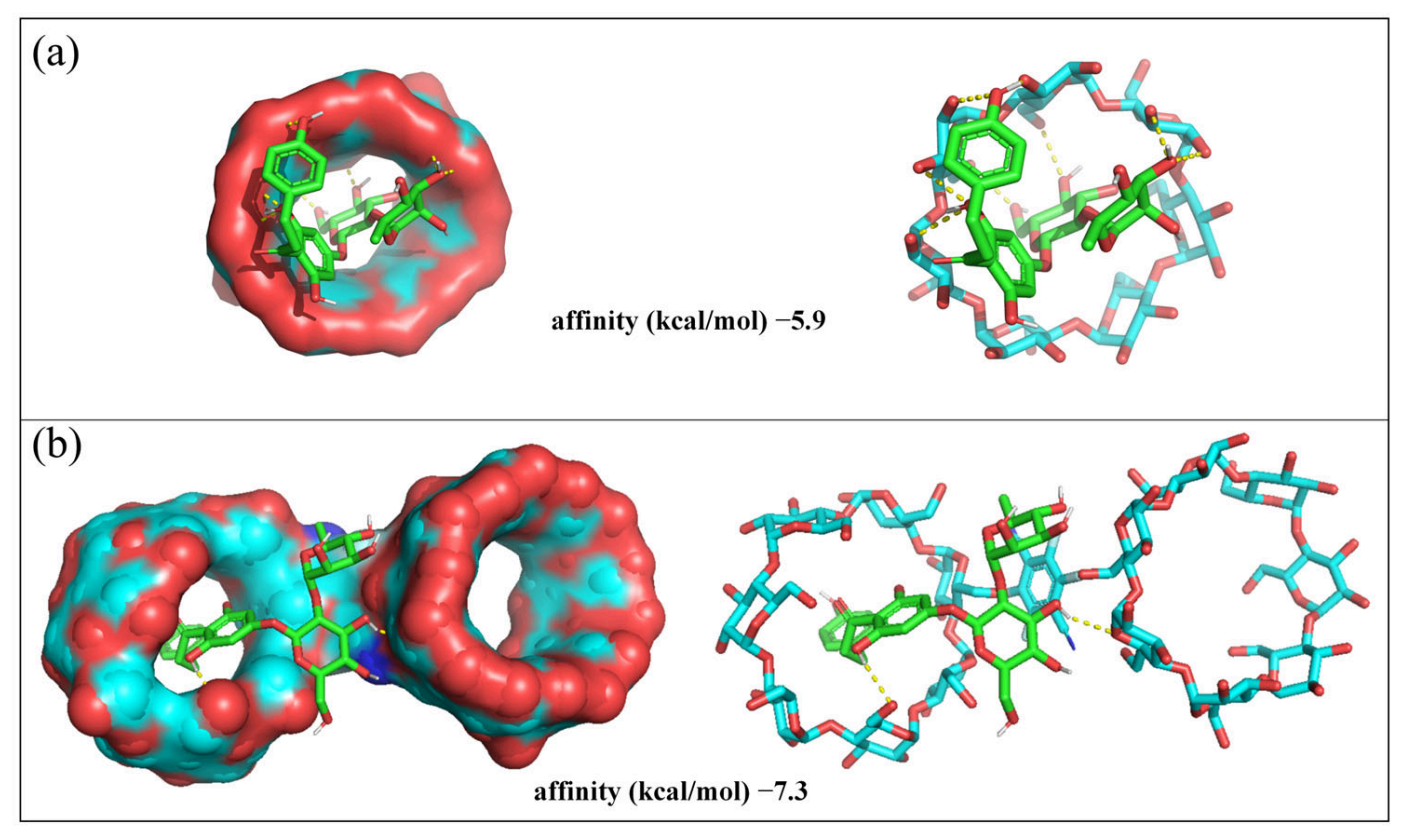
Molecular docking complex of *β*-CD monomer/*β*-CD polymer interacting with naringin (The yellow dashed line represents intermolecular hydrogen bonding forces). (**a**) *β*-CD monomer@ naringin, (**b**) two *β*-CD polymers@ naringin.

The *β*-CD polymer showed appreciable adsorption capacity and remarkably fast adsorption kinetics for naringin compared with other reported adsorbents (Table 2), highlighting its potential for large-scale debittering applications in citrus juice processing. Generally, the molecular interactions between *β*-CD and organic compounds originate from its dual structural features: a hydrophilic outer surface and a hydrophobic cavity. However, the adsorption performance and mechanisms of crosslinked *β*-CD polymers cannot be rationalized solely in terms of their monomeric building blocks. To gain deeper insight into the adsorption process, we investigated the molecular mechanisms underlying naringin adsorption onto the *β*-CD polymer using molecular docking. The binding affinities between naringin and *β*-CD monomer/*β*-CD polymer were computationally determined using AutoDock Vina 1.1.2. As shown in Figure 7a, naringin was favorably accommodated within the hydrophobic central cavity of *β*-CD monomer by hydrophobic interaction, while simultaneously forming intermolecular hydrogen bond with hydroxyl groups on the hydrophile exterior. The molecular docking revealed a binding free energy of −5.9 kcal/mol for the *β*-CD monomer–naringin complex, highlighting significantly strong affinity [54]. Furtherly, a *β*-CD polymer model was constructed by stacking two *β*-CD monomers with one PFTN molecule for docking with naringin (Figure 7b). The two dominant conformations yielded a binding free energy of −7.3 kcal/mol, indicating a substantially enhanced binding affinity compared with that of the *β*-CD monomer. Notably, in contrast to the monomer docking, naringin in this polymer model not only penetrated the hydrophobic cavity but also formed energetically favorable interactions with the peripheral hydroxyl groups of both *β*-CD units.

**Figure 8:**
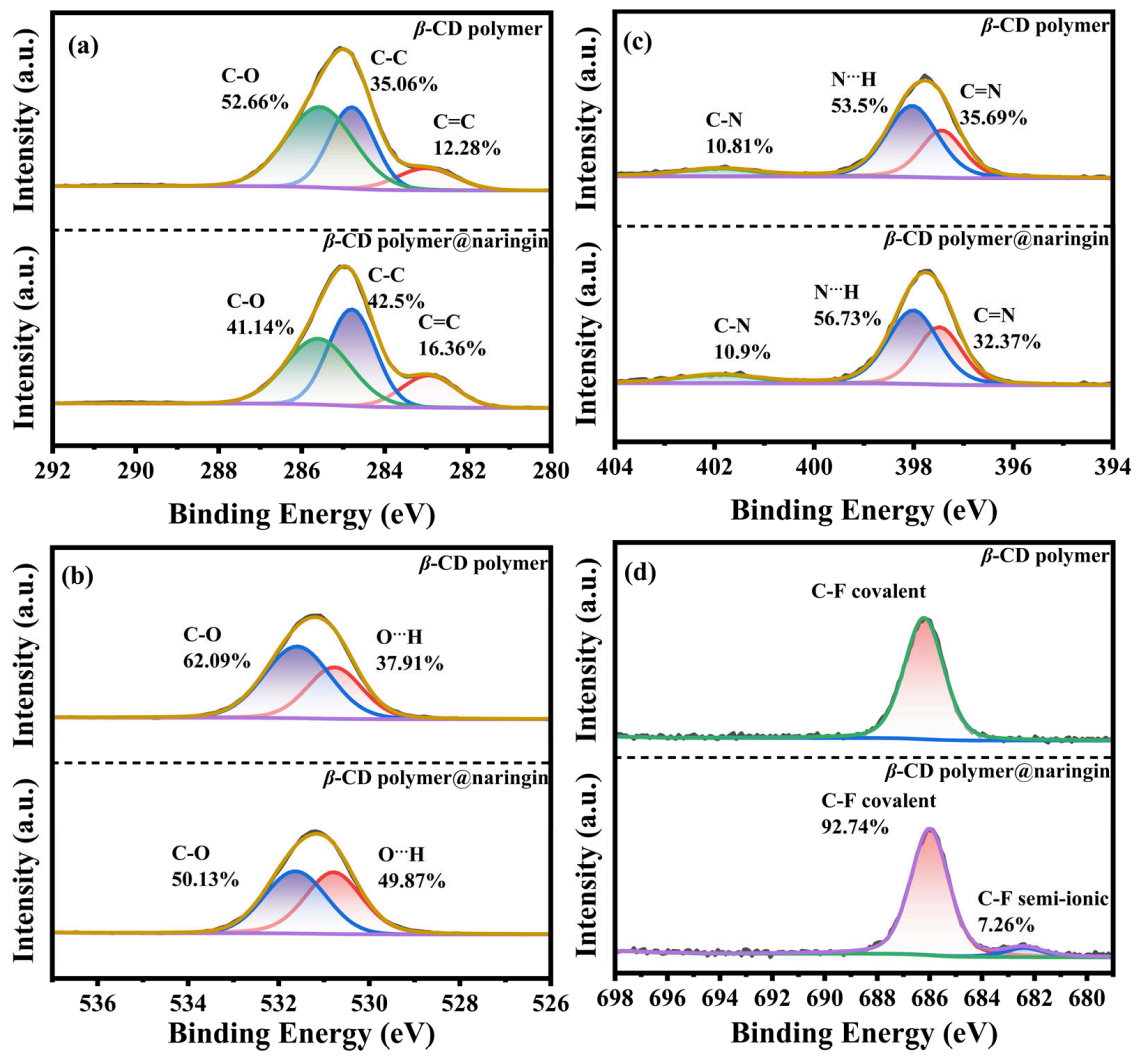
Comparative XPS analysis of *β*-cyclodextrin in naringin pre- and post-adsorption states, (**a**) C1s, (**b**) O1s, (**c**) N1s, (**d**) F1s.

The observed π–π stacking interactions between aromatic moieties were identified as a key binding mechanism. Comparative analysis of high-resolution C1s XPS spectra (Figure 8a) revealed notable changes in the chemical environment of *β*-CD polymer after naringin adsorption. XPS quantitative analysis revealed that the relative content of C–C bonds increased from 35.06% to 42.5%, while C=C bonds rose from 12.28% to 16.36% (Figure 8a), indicating enhanced π–π stacking interactions within the *β*-CD polymer–naringin complex [55,56]. Hydrogen bonding also played a significant role in the adsorption process. FT–IR analysis (Figure 2c) provided evidence for hydrogen bonding interactions, as reflected by the shifts in the –OH, C≡N groups [57]. Furtherly, this was corroborated in the high-resolution O1s spectrum (Figure 8b), where the relative content of O⋯H at 530.88 eV increased from 37.91% to 49.87% after adsorption. Meanwhile, the high-resolution N1s spectrum (Figure 8c) revealed two species of C≡N and N⋯H. Compared with the fresh *β*-CD polymer, the ratio of C≡N decreased from 35.69% to 32.37% after naringin adsorption, while the relative content of N⋯H increased from 53.5% to 56.73%, suggesting that the C≡N groups participated in hydrogen bonding with naringin. In addition, the high-resolution F1s spectrum showed notable changes after adsorption (Figure 8d), further implying that the TFPN crosslinker was involved in the adsorption mechanism. Collectively, these results confirm that hydrogen bonding, together with π–π stacking, constitutes one of the primary driving forces for naringin adsorption onto the *β*-CD polymer [56].

**Figure 9:**
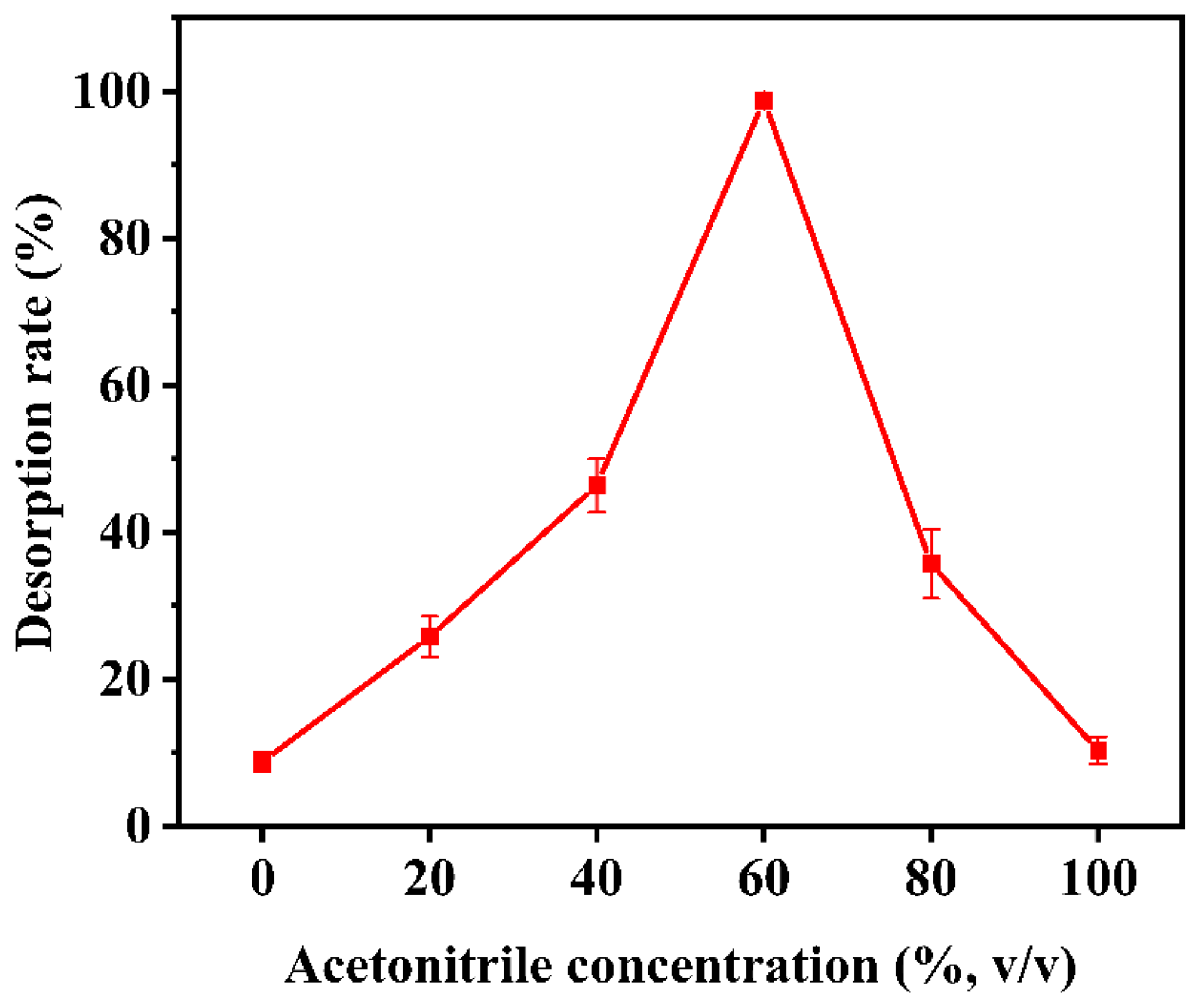
Eluent curve of on *β*-CD polymer by acetonitrile-H_2_O.

The desorption efficiency of naringin from the naringin-saturated *β*-CD polymer was systematically evaluated using six H_2_O- acetonitrile eluent mixtures with different polarity gradients. As illustrated in Figure 9, the elution profile obtained with the binary solvent system exhibited a reverse “U”-shaped curve. The desorption rate was markedly limited to 8.8% when 100% H_2_O was employed as the eluent, which can be attributed to the persistent hydrophobic interactions resisting disruption. In contrast, the optimal eluent composition of 60% acetonitrile achieved the highest elution efficiency of 98.75%, as the water-mediated hydrophilic interactions between the *β*-CD polymer and naringin were effectively disrupted, while acetonitrile simultaneously suppressed hydrophobic binding. Notably, pure acetonitrile also resulted in a low desorption efficacy of 10.3%, presumably due to the absence of water required to disrupt hydrophilic affinity. Collectively, those findings indicated that the adsorption mechanism between *β*-CD polymer and naringin is governed by hydrophobic and hydrophilic interactions [27,58].

**Figure 10:**
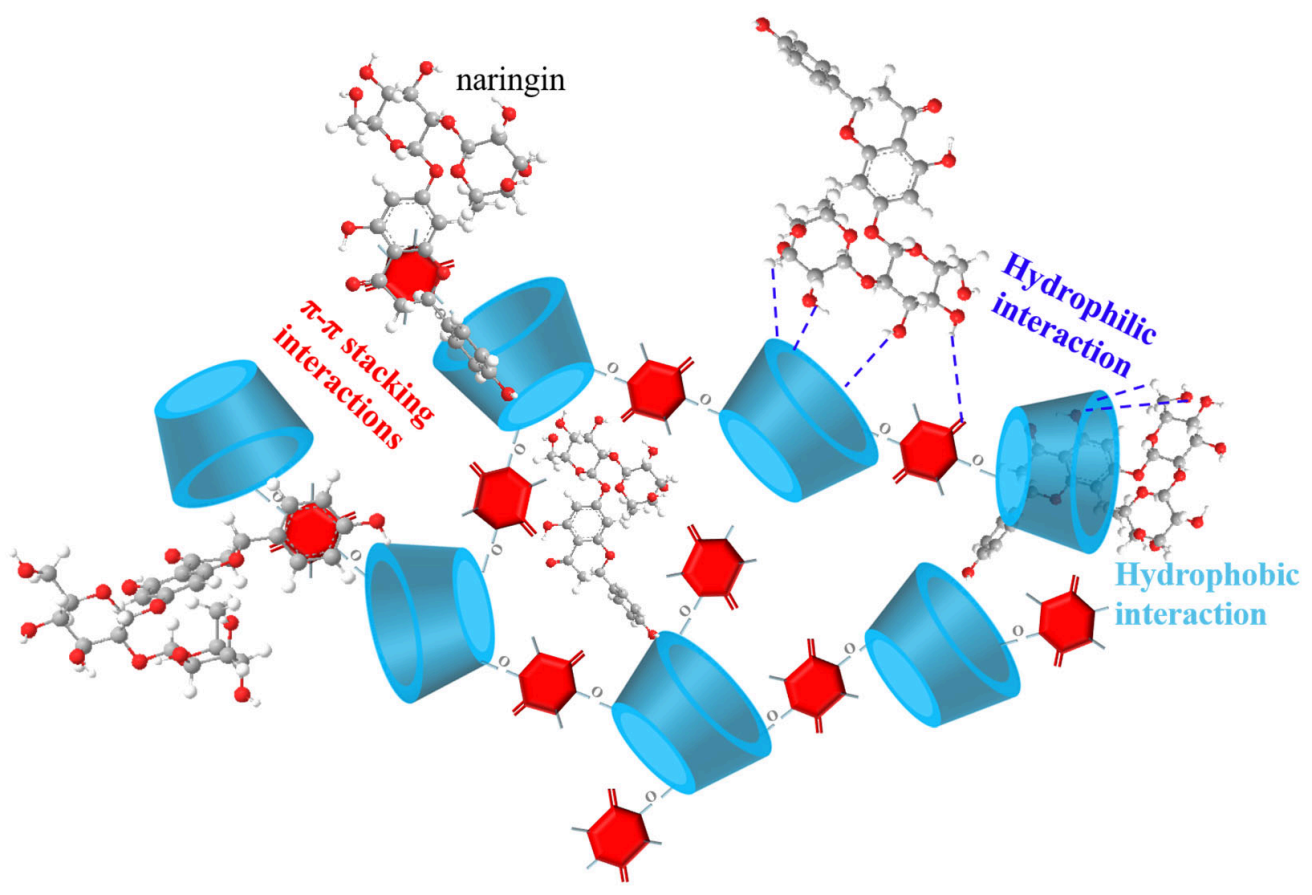
The interaction mechanism of naringin adsorption by *β*-CD polymer.

The adsorption mechanism of naringin by *β*-CD polymer could be summarized as illustrated in Figure 10. The process involves three synergistic interactions: (1) π-π stacking between the aromatic rings of TFPN and the 2-phenylchromenone moiety of naringin, (2) hydrophobic inclusion of naringin’s apolar segments within the *β*-CD cavity, and (3) hydrogen bonding among hydroxyl groups of *β*-CD and naringin, as well as the cyano groups (–CN) of TFPN. These cooperative interactions result in a dual-action system, where hydrophobic interactions govern molecular recognition, and hydrophilic interactions contribute to binding affinity and stability.

## 4. Conclusions

In this study, three types of *β*-CD polymers were synthesized and evaluated for their adsorption efficiency toward tannic acid, citric acid and naringin. Among them, the 1:2 *β*-CD polymer demonstrated the best adsorption efficiency and was therefore selected for further investigation. Static adsorption experiments indicated that the Langmuir and Toth isotherm model, as well as the PSO and Elovich kinetic models, provided superior fits compared with the Freundlich and PFO model, respectively, in terms of adsorption isotherms and kinetics. Notably, the adsorption equilibrium was achieved within 120 s, demonstrating exceptionally rapid adsorption kinetics. Single-factor experiments revealed that adsorbent dosage, initial naringin concentration and pH all significantly influenced the adsorption process, while response surface analysis further identified a more pronounced interaction between dosage and pH. Furtherly, the *β*-CD polymer exhibited excellent reusability, maintaining its original adsorption efficiency after seven consecutive regeneration cycles. Overall, the adsorption of naringin onto the *β*-CD polymer was governed by a combination of π-π stacking interactions, hydrophilic interactions and hydrophobic interactions.

## Figures and Tables

**Table 1:** Isotherm and kinetic parameters of *β*-CD polymer adsorption toward naringin.

	Isotherm			Kinetic	
Models	Parameters	Values	Models	Parameters	Values
Langmuir	q_m_ (mg/g)	24.74	PFO	q_e_ (mg/g)	19.73
	K_L_ (L/mg)	0.1266		k_1_ (s^−1^)	0.3343
	R^2^	0.9942		R^2^	0.975
	adjR^2^	0.9923		*adj*R^2^	0.9719
Freundlich	K_F_ (mg·g^−1^(L mg)^−1/n^)	5.534	PSO	q_e_ (mg/g)	20.2785
	1/n	0.3904		k_2_ (g/(mg·s))	0.0368
	R^2^	0.9767		R^2^	0.9896
	adjR^2^	0.9689		*adj*R^2^	0.9883
Toth	K_T_ (mg/g)	22.0740	Elovich	*α* (mg/(g·s))	3,008,470.98
	*a*_T_ (mg^z^·L^−z^)	17.3361		*β* (mg/g)	0.9836
	z	1.3153		R^2^	0.9978
	R^2^	0.9929		*adj*R^2^	0.9975
	adjR^2^	0.9988			

**Table 2:** The adsorption efficiency of different adsorbents for naringin.

Adsorbent	Original Concentration (mg/L)	Adsorbent Dosage (mg/L)	Adsorption Capacity (mg/g)	Adsorption Equilibrium Time	Reference
*β*-CDs based on microporous organic polymers	35	3000	1.82	360 min	[41]
MOFs based molecularly imprinted membranes	35	1000	33.32	120 min	[42]
Mesoporous carbon	100	400	Approx. 150	350 min	[43]
PVB/*β*-CD/NG/silica non-covalent imprinted composite nanofiber	20	133	7.55	35 h	[44]
Naringin-surface imprinted microspheres	58	10,000	2.87	90 min	[45]
Macroporous Resin HPD300	0.45	1200	Approx. 110	240 min	[46]
Molecular imprinting polymer/naringin	20	2	4.83	60 h	[47]
Organo-clays	500	8	45.5	720 min	[15]
Crosslinked *β*-CD polymer	10	200	20.9	120 s	This work

## Data Availability

The original contributions presented in this study are included in the article/Supplementary Materials. Further inquiries can be directed to the corresponding authors.

## Supplementary Materials

The following supporting information can be downloaded at: https://www.mdpi.com/article/10.3390/foods15142475/s1, Figure S1: Adsorption efficiency of three *β*-CD polymers toward tannic acid (absorbent dosage 1g/L, concentration 25 mg/L, 200 rpm, 2 h, the concentrations were measured using a UV–vis spectrophotometer at 270 nm by standard curve), citric acid (absorbent dosage 0.5g/L, concentration 50 mg/L, 200 rpm, 2 h, the concentration was check by acid-base titration method, based on the standard curve of the consumption of 50 mg/L NaOH) (a) adsorption rate (b) adsorption capacity; Figure S2: 3D response surface diagnostics, (a) normal probability plot of residuals, (b) correspondence diagram between residuals versus predicted values of equations, (c) plot of residuals versus predicted responses, (d) the residuals versus run number for drying time; Figure S3: 3D response surface plot (left) and the 2D contour map (right) for the interaction of the *β*-CD polymer dosage (a,b), pH (c,d), and initial naringin concentration (e,f); Table S1: Three factors and three levels of Box-Behnken design; Table S2: Box–Behnken experimental design for the optimization of naringin adsorption by *β*-CD polymer; Table S3: ANOVA data for the model of naringin adsorption by *β*-CD polymer.
