# Supplementary material for: Rapid Adsorption of Naringin from Citrus Juice by β-Cyclodextrin Polymer
Source: Foods. 2026 Jul 13;15(14):2475. doi: 10.3390/foods15142475 (PMC13409052; doi:10.3390/foods15142475)
Supplement: Supplementary file 1 [file foods-15-02475-s001.zip › foods-4388211-supplementary.pdf]

Supplementary Materials

Rapid Adsorption of Naringin from Citrus Juice by *β*-Cyclodextrin Polymer

Hai Tian [1,†] , Shuquan Lv [2,*,†], Xuepei Zhou [2], Chaohai Pang [1], Bingjun Han [1] and Yujie Feng [3,*]

[1] Analysis and Test Center, Chinese Academy of Tropical Agricultural Sciences, Hainan Provincial Key Laboratory of Quality and Safety for Tropical Fruits and Vegetables, Haikou 571101, China; tianhaischolar@163.com (H.T.); pangchaohai666@126.com (C.P.); hanbingjun6868@163.com (B.H.)

[2] School of Environmental and Biological Engineering, Wuhan Technology and Business University, Wuhan 430065, China; zhouxuepei6868@126.com

[3] Institute of Plant Protection, Hainan Academy of Agricultural Science, Research Center of Quality Safety and Standards of Agricultural Products in Hainan Academy of Agricultural Sciences, Haikou 571100, China

*Correspondence: lvshuquan@whut.edu.cn (S.L.); fengyujie238@163.com (Y.F.)

† These authors contributed equally to this work.

Supporting Information Contents

Number of sections: 2

Number of figures: 3

Number of tables: 3

Section S1. Optimized protocol of response surface optimization

Box-Behnken response surface optimization experimental design was adopted for optimizing adsorption conditions based on the results of single factor optimization. A statistically rigorous central composite design (CCD) framework was implemented using coded levels (-1, 0, +1) to analyze three operational variables: sorbent mass (X_1), initial naringin concentration in citrus juice (X_2), and solution pH (X_3). The complete experimental matrix with factor combinations and observed responses is presented in Table S1. Equilibrium adsorption capacity (Y, mg/g) served as the optimization criterion, with the predictive regression model for *β*-CD polymer performance developed through multivariate regression analysis (Eq. S1):

$$Y = \beta_0 + \sum_{i=1}^{K} \beta_i X_i + \sum_{i=1}^{K} \beta_{ii} X_i^2 + \sum_{i=1}^{K-1} \sum_{j=1}^{K} \beta_{ii} X_i X_j \qquad \text{(S1)}$$

Y denotes the response variable quantifying the *β*-CD polymer's naringin uptake capacity; β_o represents the constant coefficient; β_i, β_{ii}, and β_{ij} sequentially correspond to the linear, quadratic, and interaction coefficients; x_i and x_j signify the coded values of independent process variables as normalized dimensionless parameters.

Section S2. The detail results of response surface optimization

Experimental design and regression model: The optimized protocol for adsorption experiments was developed using Design-Expert software and the Box-Behnken Design. Three factors including absorbent dosage, initial concentration of naringin, and pH were set as independent variables, each with three levels, to achieve the maximum response through 17 groups of tests. An approximate function of adsorption capacity was calculated using Eq. (S2), where A represents the adsorbent dosage (g/L), B denotes the initial

concentration of naringin (mg/L), and C indicates pH. The Box-Behnken experimental design and the data of the β-CD polymer adsorption of naringin are shown in Table S2.

$$Y=-23.69544+63.56843A+1.41706B+9.03521C+0.531213AB-2.58586AC-0.189826BC-95.61332A^2-0.016669B^2-0.807698C^2 \quad (S2)$$

Analysis of variance (ANOVA): Statistical significance of model coefficients was assessed through analysis of variance (ANOVA) with *F*-value and *P*-values presented in Table S3. Elevated F-values coupled with lower P-values confirm the model's statistical reliability and predictive precision. Diagnostic evaluation through externally studentized residuals (Fig. S2a) revealed satisfactory agreement with theoretical normal distribution, as evidenced by linear alignment along the quantile-quantile plot's reference axis, thereby corroborating model adequacy for representing the tri-factor adsorption relationship. Residual distribution analysis (Fig. S2b) demonstrated random dispersion patterns across predicted value ranges, fulfilling the critical homoscedasticity assumption required for valid linear regression analysis. Process stability was further investigated through experimental sequence residual plotting (Fig. S2c), where data points remained within 95% confidence limits without exhibiting temporal dependencies, drift patterns, or cyclical variations - collectively indicating robust experimental control throughout the investigation. The model's predictive capacity received quantitative validation through comparative analysis (Fig. S2d) of empirical adsorption measurements (Eq. 3-derived) and theoretical predictions (Eq. 13-generated). A near-unity coefficient of determination (R^2=0.9794) and tight clustering of data points along the ideal prediction line confirm strong concordance between experimental observations and computational

simulations. This empirical-theoretical synergy validates both the methodological rigor of adsorption experiments and the mathematical sophistication of the developed *β*-CD polymer/naringin interaction model, establishing its utility for process optimization and predictive applications.

Combined effects of variables: The response surface methodology (RSM) systematically elucidated multivariate interactions governing adsorption performance through paired three-dimensional surface projections and their corresponding two-dimensional contour representations. This analytical framework maintains one parameter at central composite design levels while executing linear variation of two independent variables within prescribed experimental domains. Key graphical interpretations reveal: (1) Surface inclination magnitude in 3D projections directly reflects the parameter's influence magnitude on adsorption capacity; (2) Contour line morphology serves as an interaction strength indicator - elliptical deformation denoting significant factor interplay versus concentric circular patterns suggesting weak parameter coupling. Fig. S3 (a, b) demonstrate the *β*-CD polymer dosage-naringin concentration interdependence. Adsorption enhancement followed dose-dependent patterns (0.1-0.3 g/L) and concentration escalation, followed by capacity reduction beyond 0.3 g/L dosage thresholds. The characteristic saddle-shaped 3D surface concavity coupled with elliptical contour anisotropy confirms strong bifactorial synergy. Conversely, Fig. S3 (c, d) reveal pH-naringin concentration interactions exhibiting minimal parametric coupling, evidenced by quasi-linear contour patterns and planar 3D morphology. Adsorption enhancement showed primary dependence on naringin concentration gradients with pH fluctuations exerting negligible effects. Notably, Fig. S3 (e, f) illustrate adsorbent dosage-pH interdependencies through pronounced saddle-shaped curvature in 3D surfaces and pronounced elliptical anisotropy in contour mapping. These geometric features substantiate significant nonlinear interactions

between these process variables, suggesting pH-mediated modulation of adsorption site availability across different polymer dosages.

Process optimization and model validation: According to the defined parameters for the three variables, the optimal adsorption system achieved a maximum adsorption capacity of naringin with 18.84 mg/g under optimal conditions of 0.3 g/L adsorbent, 15 mg/L naringin in grapefruit juice, and pH 3.5. Triplicate experiments were conducted under these optimal conditions using RSM, yielding an experimental adsorption capacity of 18.84 mg/g with a standard deviation of 0.0466. The results indicate that the predicted value is close to the actual value, further demonstrating that RSM is effective for predicting the adsorption capacity of β-CD polymer toward naringin in grapefruit juice.

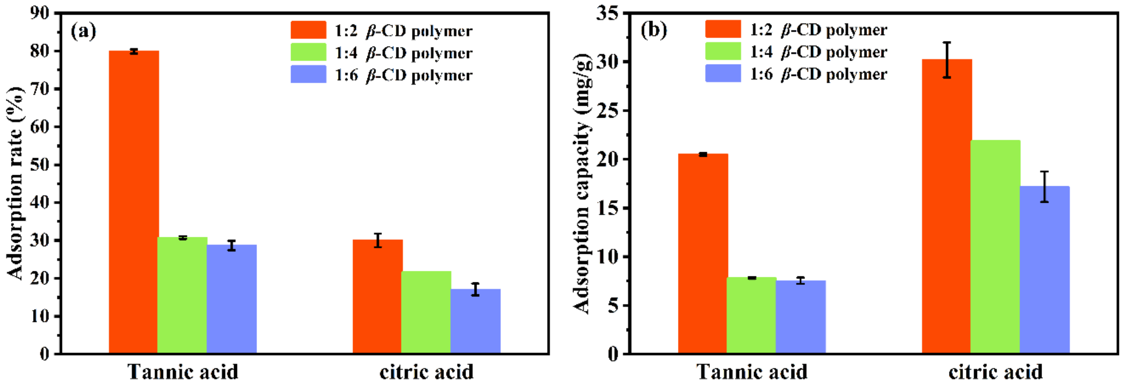

Figure S1 Adsorption efficiency of three *β*-CD polymers toward tannic acid (absorbent dosage 1g/L, concentration 25 mg/L, 200 rpm, 2 h, the concentrations were measured using a UV–vis spectrophotometer at 270 nm by standard curve), citric acid (absorbent dosage 0.5g/L, concentration 50 mg/L, 200 rpm, 2 h, the concentration was check by acid-base titration method, based on the standard curve of the consumption of 50 mg/L NaOH) (a) adsorption rate (b) adsorption capacity.

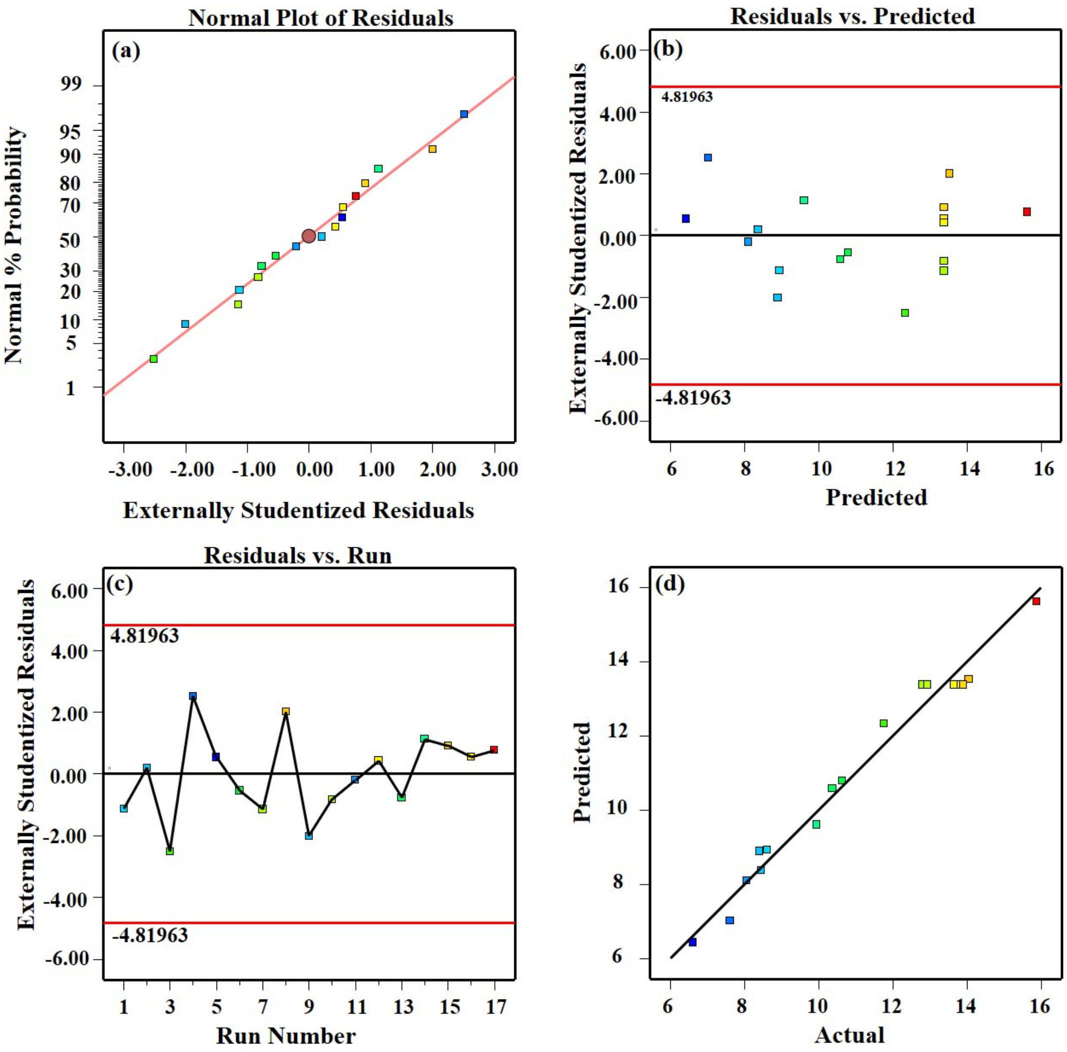

Figure S2 3D response surface diagnostics, (a) normal probability plot of residuals, (b) correspondence diagram between residuals versus predicted values of equations, (c) plot of residuals versus predicted responses, (d) the residuals versus run number for drying time.

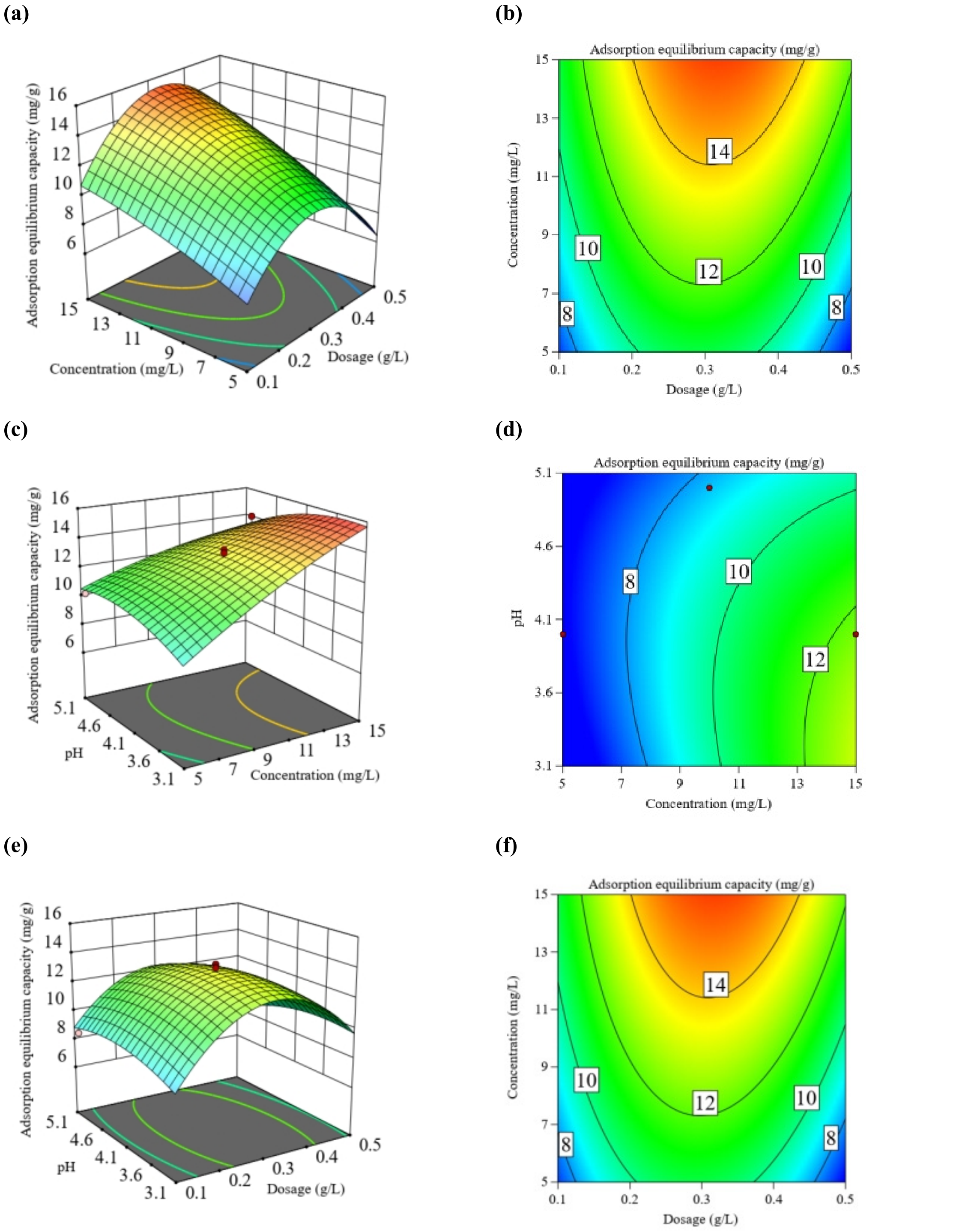

Figure S3 3D response surface plot (left) and the 2D contour map (right) for the interaction of the β-CD polymer dosage (a, b), pH (c, d), and initial naringin concentration (e, f).

Table S1 Three factors and three levels of Box-Behnken design

Factors	Levels		
	-1	0	1
Adsorbent dosage (mg/g)	0.1	0.3	0.5
pH	3	4	5
Initial concentration of naringin (mg/L)	5	10	15

Table S2 Box–Behnken experimental design for the optimization of naringin adsorption by *β*-CD polymer.

Run	Factors			Response
	Absorbent dosage (g/L)	Initial concentration (mg/L)	pH	Adsorption capacity (mg/g)
1	0.1	10	5.0	8.61
2	0.5	10	5.0	8.44
3	0.5	15	4.0	11.76
4	0.1	5	4.0	7.60
5	0.5	5	4.0	6.60
6	0.1	15	4.0	10.63
7	0.3	10	4.0	12.79
8	0.3	15	5.0	14.04
9	0.3	5	3.0	8.39
10	0.3	10	4.0	12.94
11	0.1	10	3.0	8.04
12	0.3	10	4.0	13.63
13	0.3	5	5.0	10.36
14	0.5	10	3.0	9.94
15	0.3	10	4.0	13.89
16	0.3	10	4.0	13.70
17	0.3	15	3.0	15.87

Table S3 ANOVA data for the model of naringin adsorption by *β*-CD polymer

Source	Sum of Squares	Freedom degree	Mean Square	*F*-value	*p*-value	
Model	120.93	9	13.44	37.00	< 0.0001	significant
A	0.4374	1	0.4374	1.20	0.3087	
B	46.80	1	46.80	128.89	< 0.0001	
C	0.0806	1	0.0806	0.2220	0.6518	
AB	1.13	1	1.13	3.11	0.1213	
AC	1.07	1	1.07	2.95	0.1298	
BC	3.60	1	3.60	9.92	0.0161	
A^2	61.59	1	61.59	169.61	< 0.0001	
B^2	0.7312	1	0.7312	2.01	0.1988	
C^2	2.75	1	2.75	7.56	0.0285	
Residual	2.54	7	0.3631			
Lack of Fit	1.56	3	0.5215	2.13	0.2386	not significant
Pure Error	0.9774	4	0.2443			
Cor Total	123.47	16				
Std. Dev.	0.6026		R^2		0.9794	
Mean	11.01		Adjusted R^2		0.9529	
C.V. %	5.47		Predicted R^2		0.7849	
PRESS			Adeq Precision		19.9088	
